# Molybdenum isotope fractionation by cyanobacterial assimilation during nitrate utilization and N_2_fixation

**DOI:** 10.1111/j.1472-4669.2010.00262.x

**Published:** 2011-01

**Authors:** A L Zerkle, K Scheiderich, J A Maresca, L J Liermann, S L Brantley

**Affiliations:** 1 Department of Geology and Earth System Science Interdisciplinary Center, University of MarylandCollege Park, Maryland, USA; 2 Department of Geology, University of MarylandCollege Park, Maryland, USA; 3 Department of Civil & Environmental Engineering, Massachusetts Institute of TechnologyCambridge, Massachusetts, USA; 4 Department of Geosciences, Pennsylvania State UniversityUniversity Park, Pennsylvania, USA

## Abstract

We measured the δ^98^Mo of cells and media from molybdenum (Mo) assimilation experiments with the freshwater cyanobacterium *Anabaena variabilis*, grown with nitrate as a nitrogen (N) source or fixing atmospheric N_2_. This organism uses a Mo-based nitrate reductase during nitrate utilization and a Mo-based dinitrogenase during N_2_ fixation under culture conditions here. We also demonstrate that it has a high-affinity Mo uptake system (ModABC) similar to other cyanobacteria, including marine N_2_-fixing strains. *Anabaena variabilis* preferentially assimilated light isotopes of Mo in all experiments, resulting in fractionations of −0.2‰ to −1.0‰ ± 0.2‰ between cells and media (ε_cells–media_), extending the range of biological Mo fractionations previously reported. The fractionations were internally consistent within experiments, but varied with the N source utilized and for different growth phases sampled. During growth on nitrate, *A. variabilis* consistently produced fractionations of −0.3 ± 0.1‰ (mean ± standard deviation between experiments). When fixing N_2_, *A. variabilis* produced fractionations of −0.9 ± 0.1‰ during exponential growth, and −0.5 ± 0.1‰ during stationary phase. This pattern is inconsistent with a simple kinetic isotope effect associated with Mo transport, because Mo is likely transported through the ModABC uptake system under all conditions studied. We present a reaction network model for Mo isotope fractionation that demonstrates how Mo transport and storage, coordination changes during enzymatic incorporation, and the distribution of Mo inside the cell could all contribute to the total biological fractionations. Additionally, we discuss the potential importance of biologically incorporated Mo to organic matter-bound Mo in marine sediments.

## Introduction

Molybdenum (Mo) is the most abundant transition metal in modern seawater, occurring dominantly as the molybdate anion (MoO_4_^2−^), at an average oceanic concentration of ∼105 nm (Emerson & Huested, 1991; Morford & Emerson, 1999). Molybdenum is supplied to the oceans primarily via riverine input from oxidative weathering on the continents. The dominant sinks for Mo are ferromanganese oxides deposited in oxygenated waters (accounting for ∼35% of modern marine Mo removal; Scott *et al.*, 2008), and, most significantly, conversion to particle-reactive thiomolybdates and removal by sorption onto organic matter and other reduced substrates in the presence of sulfide (e.g., McManus *et al.*, 2006).

Molybdenum has seven naturally occurring stable isotopes, with measurable mass-dependent variations that occur in natural systems (see reviews in Anbar, 2004; Anbar & Rouxel, 2007). As a result of the high concentration and long residence time of Mo in modern oceans (∼800 000 years; Collier, 1985; Emerson & Huested, 1991) seawater has a uniform isotopic composition of +2.3‰ in δ^98^Mo ((^98/95^Mo_sample_/^98/95^Mo_standard_−1) × 1000) (Barling *et al.*, 2001; Siebert *et al.*, 2003). Marine sediments, on the other hand, show a wide range of δ^98^Mo (e.g., Poulson *et al.*, 2006; Siebert *et al.*, 2006) reflecting multiple processes and sources (see review in Poulson Brucker *et al.*, 2009). The largest isotope effects to date (−3‰) have been measured during adsorption of Mo to Mn-oxides and Fe(oxyhydr)oxides (Siebert *et al.*, 2003; Barling & Anbar, 2004; Wasylenki *et al.*, 2006; Goldberg *et al.*, 2009), concentrating isotopically-light Mo in ferromanganese sediments deposited in oxic settings (e.g., Barling *et al.*, 2001). In contrast, in euxinic basins with free sulfide in the water column, Mo is nearly completely removed into the sediments such that no fractionation from the seawater value is expressed (Barling *et al.*, 2001; Arnold *et al.*, 2004; Nägler *et al.*, 2005).

The variation in Mo removal processes and associated isotopic signatures under different redox settings formed the basis of early models of Mo isotopes in ancient black shales as a paleoredox proxy (e.g., Arnold *et al.*, 2004). In this simple model, euxinic sediments were assumed to capture the δ^98^Mo of overlying seawater, reflecting the proportion of the global burial of Mo in oxic vs. euxinic sinks. Recent measurements of sedimentary Mo isotope values in ‘suboxic’ environments (defined here as having low bottom-water O_2_, but lacking free sulfide in the water column) show δ^98^Mo values between oxic and euxinic settings, complicating this simple interpretation (e.g., Poulson Brucker *et al.*, 2009). These low O_2_ sediments have δ^98^Mo values that are depleted in ^98^Mo from overlying seawater by ∼0.7–2‰ (Poulson *et al.*, 2006; Siebert *et al.*, 2006). The dominant controls on the fractionations produced in these environments are not well constrained, but could reflect Fe-Mn-S systematics (Barling *et al.*, 2001; Siebert *et al.*, 2003; Reitz *et al.*, 2007; Wasylenki *et al.*, 2008), transitions between oxic and sulfidic Mo species (Tossel, 2005), or interactions with organic matter (e.g., McManus *et al.*, 2006).

A significant amount of Mo is associated with organic matter in marine systems, both incorporated into cells and sorbed to organic particles in the water column (e.g., Tribovillard *et al.*, 2004). Biologically, Mo is an essential micronutrient for all three domains of life, serving as a cofactor for enzymes involved in carbon, nitrogen, and sulfur metabolisms (Frausto da Silva and Williams, 2001). Most significantly, Mo plays a prominent role in enzymes involved in the nitrogen cycle (see reviews in Zhang & Gladyshev, 2008; Glass *et al.*, 2009), acting as metal cofactor for the primary enzyme utilized in nitrate assimilation (nitrate reductase) and for one component of the dominant nitrogenase enzyme complex utilized in nitrogen fixation (dinitrogenase) (Miller & Eady, 1988; Howard & Rees, 1996). Dinitrogenases containing Mo have been isolated from numerous prokaryotes, including both bacteria and archaea, some of which are fungal and plant endosymbionts (see review in Howard & Rees, 1996). All known N_2_-fixing organisms (diazotrophs) utilize a dinitrogenase with an iron-molybdenum (Fe-Mo) cofactor, containing 2 moles of Mo per mole of enzyme complex (Howard & Rees, 1996). When Mo is scarce, some organisms can produce two homologous alternative dinitrogenases, containing either an iron-vanadium cofactor or a cofactor containing only Fe (Eady, 1996). The alternate enzymes have been found only secondarily to the Mo-containing dinitrogenase in a subset of organisms, and are significantly less efficient than the primary enzyme (Joerger & Bishop, 1988; Miller & Eady, 1988). Some diazotrophs, including *Anabaena variabilis*, can also produce a different Fe-Mo-dependent dinitrogenase under anoxic conditions (Thiel *et al.*, 1995, 1997; Thiel & Pratte, 2001).

Biological fractionations of Mo are not well constrained. Previous work has focused on cultures of the N_2_-fixing soil bacterium *Azotobacter vinelandii* (Liermann *et al.*, 2005; Wasylenki *et al.*, 2007). One group reported fractionations during Mo assimilation in cultures of the marine N_2_-fixing cyanobacterium *Trichodesmium* sp. IMS 101, but these results were only published in a conference abstract (Nägler *et al.*, 2004) and have not been expanded upon since. These studies have demonstrated that bacteria can concentrate the light isotopes of Mo during uptake, producing measurable fractionations (Table 1). However, *Azotobacter vinelandii* has two unique or rare biochemical strategies for the uptake and storage of Mo, including the production of Mo-chelating ligands, or ‘molybdophores’, for the scavenging of Mo in terrestrial systems (Liermann *et al.*, 2005; Bellenger *et al.*, 2008), and the possession of a rare Mo storage protein (MoSto), which can store up to ∼80 atoms of Mo as a Mo-oxide aggregate (Pienkos & Brill, 1981; Fenske *et al.*, 2005; Schemberg *et al.*, 2007, 2008). *Azotobacter vinelandii* also utilizes a periplasmic Mo-binding protein ModA, which is part of the high-affinity Mo uptake system ModABC, that shows weak sequence similarity but similar structure to the periplasmic Mo-binding proteins of freshwater cyanobacteria (Zahalak *et al.*, 2004). The fractionations produced by *Azotobacter vinelandii* have been linked to molybdophore chelation and/or to binding by this ModA protein (Liermann *et al.*, 2005; Wasylenki *et al.*, 2007), and therefore could differ significantly from fractionations produced in aqueous organisms with different uptake strategies. In order to extrapolate biological fractionations to aqueous sedimentary systems, it is necessary to further examine fractionations associated with Mo assimilation in aqueous organisms, particularly in cyanobacteria, which are the dominant source of fixed N to the modern biosphere (Capone *et al.*, 1997; Zehr *et al.*, 2001; Montoya *et al.*, 2004), and have likely been fixing N_2_ since early in geologic time (e.g., Kasting & Siefert, 2001; Tomitani *et al.*, 2006).

**Table 1:** Compilation of previous studies of biological Mo isotope fractionations (in ‰), along with this study (± analytical or given 2σ). Also shown are the N source, initial [Mo] (when reported), Mo source (glass or aqueous Mo), growth phase (as reported), and the number of individual analyses reported (not including duplicates) (*n*)

Organism	Type	N source	[Mo], source	Growth phase	δ^98^Mo fractionation	*n*	Ref.
*Trichodesmium* sp.	Marine cyanobacterium	N_2_	Not given, [Mo]_aq_	Early, late	−0.5, −0.1 ± 0.1	2	1
*Azotobacter vinelandii*	Soil bacterium	NH_3_	1.5 μm, glass	Not given	−0.8 ± 0.4*	5	2
*Azotobacter vinelandii*	Soil bacterium	NH_3_, N_2_	∼1 μm, [Mo]_aq_	Not given	−0.5 ± 0.2*	11	3
*Anabaena variabilis*	Fw cyanobacterium	NO_3_^−^	1.6 μm, [Mo]_aq_	Late exp., stationary	−0.3, −0.3 ± 0.2	7	4
*Anabaena variabilis*	Fw cyanobacterium	N_2_	1.7 μm, [Mo]_aq_	Exp., stationary	−0.9, −0.5 ± 0.2	7	4

1, Nägler *et al.*, 2004 (reported only in an abstract from conference proceedings); 2, Liermann *et al.*, 2005; 3, Wasylenki *et al.*, 2007, 4. This study

* Values converted from δ^97/95^Mo to δ^98/95^Mo, assuming δ^97/95^Mo ∼ 2/3 δ^98/95^Mo

In this study, we examined the fractionations associated with Mo assimilation during nitrate reduction and N_2_ fixation in cultures of the freshwater cyanobacterium *Anabaena variabilis* ATCC 29413. *Anabaena variabilis* is a filamentous heterocystous cyanobacterium. Heterocystous cyanobacteria are relatively rare in the modern oceans; however, several lines of evidence point to shared biochemical pathways for Mo uptake and utilization in marine and freshwater cyanobacteria. *Anabaena variabilis* utilizes a Fe-Mo dinitrogenase homologous to that of marine cyanobacteria when grown aerobically in the presence of Mo (e.g., Thiel, 1993), and a homologous Mo-dependent nitrate reductase during nitrate utilization (Zahalak *et al.*, 2004). The *nifDK* gene encoding for the dinitrogenase (Fe-Mo) protein of *A. variabilis* clusters together with other cyanobacterial *nifDK* genes sequenced, including the marine N_2_-fixing cyanobacterium *Trichodesmium* sp. (Dominic *et al.*, 2000). We examined genes for the ModABC high-affinity Mo uptake system in *A. variabilis*, and demonstrate that these genes similarly cluster together with those of marine N_2_-fixing cyanobacteria. We then examine fractionations in Mo isotopes during nitrate reduction and N_2_ fixation in this organism as a first step in quantifying the biological fractionations expected to be produced in aqueous sedimentary systems.

Our results indicate that this organism can produce fractionations similar to or larger than those of the soil bacterium *Azotobacter vinelandii* (as large as −1.0‰), particularly when fixing N_2_ under growth conditions when N is the only limiting nutrient. Furthermore, these fractionations vary both with the N source utilized and with the growth phase sampled (for N_2_ fixation), indicating a fractionation mechanism (or mechanisms) more complex than a simple kinetic effect during cellular Mo uptake. We utilize a metabolic model of the Mo physiology in a first attempt to elucidate the mechanism(s) for and potential limits of Mo isotope fractionation during biological assimilation.

## Methods

### ModABC sequence alignments

We compared genes for ModA, the periplasmic Mo-binding protein of the ModABC transport system, from *A. variabilis* with 53 ModA amino acid sequences that were selected from the NCBI-nonredundant (NCBI-nr) database, including 13 cyanobacterial sequences and representative sequences from a variety of other bacterial taxonomic groups. Bacterial ModA proteins that have been biochemically, genetically, or structurally characterized were included (see Table S1, Supporting information). Some archaeal ModA proteins have been characterized; these sequences were excluded from the tree because they could not be aligned reliably with the bacterial sequences. The sequences were aligned with clustalw and the alignment was manually adjusted. A neighbor-joining phylogenetic tree (Saitou & Nei, 1987) was calculated in MEGA (Tamura *et al.*, 2007) using the Dayhoff model for amino acid substitution (Schwarz & Dayhoff, 1979), and 500 bootstrap replicates. The predicted amino acid sequence from the *A. variabilis* fused *modBC* gene (encoding the other two components of the ModABC transport system) was used in a blastp search (Altschul *et al.*, 1990) against the NCBI-nr database and the lengths of the alignments were plotted along the *A. variabilis* ModBC sequence.

### Experimental methods

*Anabaena variabilis* str. ATCC 29413 was grown in a modified version of medium 819, containing the following components per liter of Milli Q H_2_O: 0.04 g K_2_HPO_4_, 0.075 g MgSO_4_·7H_2_O, 0.036 g CaCl_2_·2H_2_O, 0.02 g Na_2_CO_3_, 6 mg citric acid, 1 mg EDTA, and 1 mL of Trace Metal Mix A5 [with 2.86 g H_3_BO_3_, 1.81 g MnCl_2_·4H_2_O, 0.222 g ZnSO_4_·7H_2_O, 0.079 g CuSO_4_·5H_2_O, and 49 mg Co(NO)·H_2_O per liter of Milli Q H_2_O]. We additionally included 10% fructose as a carbon source to stimulate growth and N_2_ fixation (Haury & Spiller, 1981). Separate solutions of Na_2_MoO_4_·2H_2_O and Fe-citrate were added to final [Mo] of 1.6 ± 0.1 μm and [Fe] of ∼20 μm, measured by inductively coupled plasma mass spectrometry (ICP-MS) (Mo & Fe) and isotope dilution (Mo), as described below. *Anabaena variabilis* strains in which *modBC* had been inactivated transported Mo at 10 μm but did not transport Mo at 1 μm (Zahalak *et al.*, 2004), suggesting that the ModABC transport system is utilized in all of the Mo conditions studied here.

For nitrate utilization experiments, NaNO_3_ was added to an excess nitrate concentration of ∼18 mm. Cultures were prepared using standard aseptic techniques, in acid-washed polycarbonate vessels, and grown in a shaking light box under atmosphere with constant light (∼70 μE m^−2^ s^−1^) and optimal pH (7.1) and temperature (33 °C) (e.g., Zahalak *et al.*, 2004). Stock cultures of nitrate-utilizing and N_2_-fixing cultures were maintained separately to ensure consistency of nitrogen source. Growth was tracked by optical density measurements at 600 nm and calibrated to counts of individual cells within filaments using a standard DAPI (4′,6-diamidino-2-phenylindole) staining. Robust growth curves for the organism grown under the conditions of this study were established from growth of over 40 individual cultures prior to experiments. Parallel cultures were analyzed for nitrogenase activity in triplicate, using the standard acetylene-ethylene technique (Dilworth, 1967; Schollhorn & Burris, 1967). Experiments were started by inoculating 2–5% of cells from stationary phase, resulting in a negligible transfer of biomass Mo to the start of the experiment. Four sets of experiments were run in 200–300 mL batches in triplicate with blanks containing medium only.

Experiments were processed on a time series after 5 and 6 days (with nitrate) or 6 and 9 days (fixing N_2_). Select N_2_ fixation experiments were additionally split for C:N and δ^15^N ratios, analyzed using a Costech/Thermo-Finnigan Delta Plus XP coupled elemental analyzer, continuous flow, isotope ratio mass spectrometer (EA-CF-IRMS), as described in a companion study (Zerkle *et al.*, 2008). Controls were processed in a manner identical to experiments. Cells were first concentrated via centrifugation, rinsed several times with Milli Q water and 1 mm EDTA to remove weakly sorbed metals, transferred to Teflon Savillex vials, and digested in ultrapure HNO_3_ and HF. Cells viewed under light microscopy after centrifugation and rinsing showed no signs of significant lysis. Media were filtered through a pre-sterilized filtration apparatus and acidified with ultrapure HNO_3_ and HF. Media and digested cell pellets were initially screened for Mo concentrations by ICP-MS at the Materials Characterization Laboratory at The Pennsylvania State University (estimated uncertainties were ±5% for media and ±10% for cell pellets). Experimental blanks that were treated identically yielded Mo below analytical detection (<0.2 μg). Final total Mo concentrations for processed media and cells were calculated from isotope dilutions (as described below) with an estimated uncertainty of <1%.

### Isotope analyses

Samples were processed and analyzed for Mo isotopes at the University of Maryland, following methods outlined in Scheiderich *et al.* (2010). Select media samples were split and processed separately to ensure internal consistency of methodology. Samples were acidified with concentrated, quartz-distilled HNO_3_ and weighed in open Teflon Savillex beakers using an evaporation-correction technique. An appropriate quantity of ^97^Mo–^100^Mo double spike was added (based on screened concentrations) and weighed by evaporation correction. The samples were then closed and heated on a 90 °C hot plate for ∼6 h to equilibrate the sample and spike Mo. The resulting solution was dried down with concentrated quartz-distilled HCl and ultra-pure HClO_4_ and re-dissolved in 6 m HCl in preparation for ion-exchange chromatography.

A three-column chromatographic separation was used to purify samples. The first and last columns were anion separations using AG 1x8 resin, and an elution sequence modified from Pietruszka *et al.* (2006). Briefly, the sample was loaded in 6 m HCl, rinsed with 6 m HCl, then 0.01 m HCl/0.1 m HF, and finally Mo was eluted with 1 m HCl. The second column was a cation-exchange separation, using AG 50Wx8, with the sample loaded and eluted in 1.4 m HCl, as described in Scheiderich *et al.* (2010). Just prior to analysis, the separated Mo was dissolved in an appropriate amount of 2% ultra-pure nitric acid and refluxed. Digestion and column blanks were assessed by passing a known amount of ^97^Mo spike through the digestion and column separation chemistry, and were typically less than 3 ng, based on repeat analyses.

Isotopic measurements were made in static mode using a Nu Instruments (Wrexham, North Wales, UK) multi-collector ICP-MS, using either an Apex IR (Elemental Scientific Inc., Omaha, NE, USA) nebulizer with an uptake rate of ∼50 μL min^−1^, or a Scott Double Pass Peltier cooled spray chamber with an uptake rate of ∼1 mL min^−1^. A gain calibration was run each day that measurements were made. After a minimum of 2 h warm-up time, the instrument was tuned to at least 2 V on ^98^Mo for all measurements. A single measurement consisted of 60 ratios, with a zero cycle at half-mass after every block of 15 ratios. Zirconium and Ru, which overlap the Mo mass spectrum, were monitored on one isotope each (90 and 99, respectively) to ensure that no direct interferences were occurring from these elements. Instrument performance was monitored on a daily basis by repeatedly measuring an in-house Johnson-Matthey Company (London, England) SpecPure® Mo plasma standard (Stock #35758, Lot #013186S), and calculating an internal fractionation factor for ^98/95^Mo. The fractionation factor was then used to calculate internal fractionation-corrected ratios for ^92/95^Mo, ^97/95^Mo, and ^100/95^Mo. These ratios were then compared, by means of an epsilon calculation, to the ‘accepted value’ for the given ratio. No sample measurements were made until the epsilon value was within ±1 epsilon unit of the accepted value.

Raw isotope ratios were deconvoluted using a modified version of the Siebert *et al.* (2001) method. Delta values were calculated by comparing the deconvoluted sample ^98/95^Mo ratio to the ^98/95^Mo ratio for an in-house Johnson-Matthey Company SpecPure® Mo plasma standard, using the standard delta notation: δ^98^Mo = ((^98/95^Mo sample/^98/95^Mo standard)−1) × 1000. A fractionation-corrected ^98/95^Mo ratio for the standard was determined by measuring double-spiked aliquots and reducing the raw ratios. This procedure provides an additional check on long-term instrument performance with respect to Mo isotopes, through comparison of δ^98^Mo values for the Mo standard. The SpecPure® Mo plasma standard is frequently used in the literature as the standard reference to calculate a delta value for seawater (+2.3‰, Siebert *et al.*, 2003). Long-term external reproducibility of δ^98^Mo measurements is based on replicate processing and multiple analyses of two sediment reference materials SDO-1 and New Albany Shale, which have δ^98^Mo = +0.88 ± 0.19‰, 2σ, *n* = 54, and +0.31 ± 0.20‰, 2σ, *n* = 27, respectively. SDO-1 has been measured by other workers (Barling *et al.*, 2001; Wasylenki *et al.*, 2008; Poulson Brucker *et al.*, 2009) and our data compare favorably. Any small differences are likely because of the use of variations in isotopic composition of batches of SpecPure® Mo. Internal precision of ratio measurements is better than ±0.01% (2σ).

## Results

**Fig. 1:**
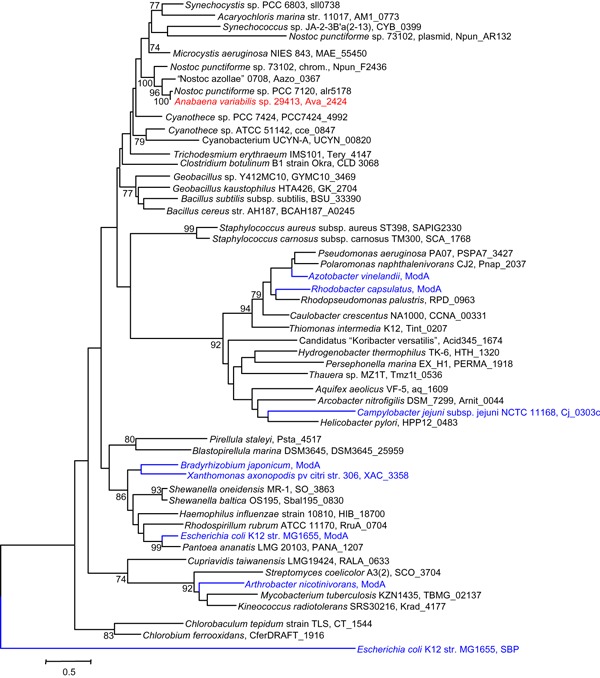
Neighbor-joining tree of ModA proteins. *Anabaena variabilis* ModA is in red; sequences that have been shown to be Mo-binding proteins are in blue. Fifty-three protein sequences were aligned; bootstrap values (percent of 500 replicates) greater than 70% are shown. The ModA from *A. variabilis* clearly groups with other cyanobacterial proteins.

The ModA amino acid sequence (encoding for the periplasmic Mo-binding protein of the ModABC transport system) from *A. variabilis* was aligned with ModA sequences from a variety of microbial species and used to calculate a distance-based phylogenetic tree (Fig. 1). Bootstrap values greater than 70% are shown next to the branches. The *A. variabilis* ModA (red) groups with very high bootstrap support with other ModA sequences from the Nostocales, a sub-group within the cyanobacterial ModA clade. The other biochemically characterized ModA sequences, including *Azotobacter vinelandii* (blue lines), clearly group separately from the cyanobacterial sequences, including modern marine N_2_-fixing cyanobacteria (e.g., *Trichodesmium erythraeum* IMS101).

The other components of the Mo uptake system include Mod B, the transmembrane component, and ModC, which provides energy on the cytoplasmic side of the membrane. The *modBC* gene from *A. variabilis* is a fusion; in most microbes, *modB* and *modC* are separate genes. The predicted amino acid sequence of ModBC in *A. variabilis* was used as the query in a blastp search against the NCBI-nr database. The only species that had open reading frames that aligned with the full-length ModBC protein were either cyanobacterial or algal (Fig. S1, Supporting information). Some cyanobacterial genomes encode genes whose predicted products only align partially with the *A. variabilis* ModBC sequence. However, most of these genomes also encode a full-length ortholog of ModBC, and the shorter homolog is annotated as a sulfate transporter. Several Firmicutes have orthologs of ModB and ModC, but it is clear that the *A. variabilis* ModBC sequence aligns with two different open reading frames in all of these species (Fig. S1, Supporting information).

**Fig. 2:**
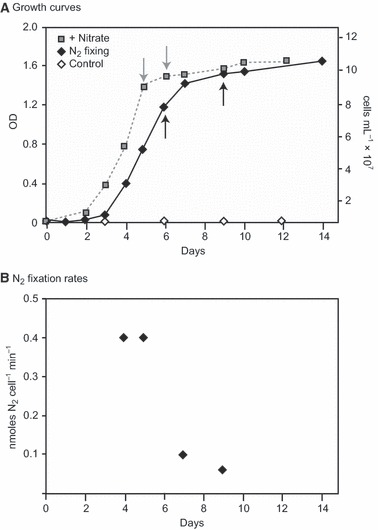
Composite growth curves (A) and N_2_ fixation rates (B) for *Anabaena variabilis*. The experiments reported here were processed and analyzed for isotopes at times indicated by arrows on the plot.

Growth curves and N_2_ fixation rates for *A. variabilis* are shown in Fig. 2. Note that N_2_-fixing organisms never reach a true ‘exponential’ growth phase, presumably because growth was limited by the diffusion of N_2_ into the cells (e.g., Allen & Arnon, 1955), but do exhibit log-linear growth. We refer to this growth phase as ‘exponential’ here in keeping with standard terminology. The cultures then enter a stationary phase, where growth and metabolic activities continue, but very slowly. In these experiments stationary phase occurred when the culture was limited by organic carbon. Identical batch experiments conducted under autotrophic growth conditions continued in accelerated growth for at least 10 days when CO_2_ was continuously added. Nitrogen fixation rates varied from 0.4 to 0.06 nmoles N_2_ per cell per minute. N_2_ fixation continued during early stationary phase, albeit at lower rates (Fig. 2B). No ethylene production was measured in cultures grown with nitrate.

Biomass C:N ratios in N_2_-fixing organisms were measured to equal 5.2–6.4, with δ^15^N values of −1.3 to −2.0‰ relative to atmospheric N_2_. We calculated higher cellular Mo levels in N_2_-fixing cultures (from 0.4 to 1.5 fg Mo per cell) than in cultures grown with nitrate (from 0.1 to 0.6 fg Mo per cell) based on measured Mo concentrations in digested cell pellets and cell counts. These cellular Mo levels are similar to Mo levels measured in marine N_2_-fixers (Tuit *et al.*, 2004); however, we estimate large errors for these quantities based on the uncertainty associated with cell counts.

The measured Mo concentration of the starting media was 1.6 ± 0.1 μm. Total masses of Mo processed for isotope analyses of media and cells, in μg, are listed in Table 2. These quantities differ between experiments because different volumes of sample were processed (some samples were split for parallel analyses) but all samples analyzed satisfied mass balance (initial media Mo = final media Mo + cellular Mo).

**Table 2:** Experimental results. Values of δ^98^Mo, *α*, and *ε* are calculated with equations listed in the text, and given in ‰. Media samples split, processed, and analyzed separately differed by less than long-term analytical reproducibility (<0.2‰)

Expt.	N source	Expt. Duration (days)	Mo_media_ (μg)	Mo_cells_ (μg)	*F*_cells_	δ^98^Mo_media_	δ^98^Mo_cells_	*α*_cells–media_	*ε*_cells–media_
Blank	Nitrate		12.6	<0.2		0.14			
Blank	N_2_		15.7	<0.2		0.14, 0.11			
1A	Nitrate	5	23.8	2.0	0.08	0.13, 0.13	−0.29	0.99960	−0.40
1B	Nitrate	5	29.4	1.8	0.06	0.17, 0.15	−0.19	0.99966	−0.34
1C	Nitrate	5	36.3	0.5	0.01	0.07, 0.12	−0.11	0.99981	−0.19
Mean ± standard deviation								0.99969	−0.3 ± 0.1
2A	Nitrate	6	18.7	4.1	0.18	0.19	−0.20	0.99965	−0.35
2B	Nitrate	6	12.7	3.1	0.20	0.15, 0.25	−0.17	0.99967	−0.33
2C	Nitrate	6	20.2	2.4	0.11	0.07	−0.10	0.99984	−0.16
Mean ± standard deviation								0.99972	−0.3 ± 0.1
3A	N_2_	6	40.9	4.9	0.11	0.47, 0.38	−0.57	0.99906	−0.94
3B	N_2_	6	36.8	1.5	0.04	0.29, 0.21	−0.54	0.99923	−0.77
3C	N_2_	6	27.1	4.8	0.15	0.37, 0.45	−0.66	0.99901	−0.99
Mean ± standard deviation								0.99910	−0.9 ± 0.1
4A	N_2_	9	26.0	7.0	0.21	0.26, 0.39	−0.32	0.99943	−0.57
4B	N_2_	9	25.2	4.8	0.16	0.25, 0.27	−0.25	0.99941	−0.59
4C	N_2_	9	18.9	7.8	0.29	0.30	−0.12	0.99965	−0.35
Mean ± standard deviation								0.99949	−0.5 ± 0.1

Measurements of δ^98^Mo for media and cell pellets are also listed in Table 2. We calculated the value of *α*_cells–media_, the fractionation factor between media and cells, by solving a derived Rayleigh fractionation equation:


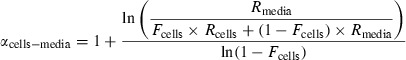
(1)

Here *F*_cells_ is the fraction of total Mo in the cells at sampling (= cellular Mo/(cellular Mo + final media Mo)), and *R*_*i*_ is the measured isotope ratio in the media or cells as indicated by subscript *i*, ^98/95^Mo_sample_/^98/95^Mo_standard_. We report isotope fractionations between cells and media as ε values, defined by:


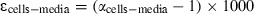
(2)

This quantity is comparable to the ΔMo (≍δ^98^Mo_media_−δ^98^Mo_cells_) used in previous studies (e.g., Wasylenki *et al.*, 2007).

The results show that cells preferentially accumulated the lighter isotopes of Mo, resulting in ɛ_cells–media_ values of −0.2 to −1.0‰ (Table 2). The fractionations were internally consistent within experiments, but varied between experiments with N source utilized and during growth phases for N_2_ fixation. When utilizing nitrate as an N source, fractionations of −0.3 ± 0.1‰ (mean ± standard deviation between experiments) were consistently observed. During N_2_ fixation, fractionations of −0.9 ± 0.1‰ were observed during exponential growth, while fractionations of −0.5 ± 0.1‰ were observed during stationary phase. A similar growth dependence for fractionation was also reported for an N_2_-fixing marine cyanobacterium (Nägler *et al.*, 2004).

## Discussion

The −0.2 to −1.0‰ fractionations produced in experiments with *A. variabilis* extend the magnitude of biological Mo fractionations previously reported for a soil bacterium (Liermann *et al.*, 2005; Wasylenki *et al.*, 2007) and a marine cyanobacterium (Nägler *et al.*, 2004) (Table 1). Although the data is very limited, these fractionations also vary between the nitrogen metabolisms tested and with growth phase during N_2_ fixation.

Previous workers have attributed biological Mo isotope fractionations to (i) coordination changes during uptake with a chelating ligand; (ii) sorption of Mo to the cell surface; or (iii) a simple kinetic isotope effect associated with irreversible Mo transport (Liermann *et al.*, 2005; Wasylenki *et al.*, 2007). The strain of *A. variabilis* examined here is not known to produce any metal-scavenging ligands, though a similar strain (PCC 7937) can produce a high-affinity siderophore for Fe scavenging under Fe-depleted conditions (Kerry *et al.*, 1988). Furthermore, all experiments in this study were conducted under Fe- and Mo-replete conditions, when no ligand production would be expected to occur. We therefore consider coordination changes during uptake with a secreted chelating ligand an unlikely source of the fractionations.

We rinsed the cells with EDTA to remove weakly sorbed metals, but it is nonetheless possible that significant amounts of Mo could have sorbed to Fe- and/or Mn-oxides precipitates if they were present on cell walls (Tovar-Sanchez *et al.*, 2003). However, the fractionations are inversely correlated with culture density (Fig. 2; Table 2), which is inconsistent with an adsorption mechanism for fractionation. Finally, a single kinetic isotope effect associated with Mo transport would not differ between nitrogen metabolisms, because the high-affinity Mo transporter ModABC is utilized during all conditions tested here, including both nitrate reduction and N_2_ fixation (Zahalak *et al.*, 2004). Instead, the pattern in δ^98^Mo fractionations we observe suggests a more complex mechanism or mechanisms for fractionation. This mechanism has to explain the differences in fractionations produced with different N sources and with progressive growth during N_2_ fixation.

To explore the possible mechanism(s) for Mo isotope fractionation we constructed a reaction network model of the cyanobacterial Mo metabolism based on the biochemical pathways for Mo utilization during nitrate reduction and N_2_ fixation. This is a common approach that has been used to examine biological fractionations in many different isotope systems, including carbon, nitrogen, and sulfur (e.g., Harrison & Thode, 1958; Rees, 1973; Cypionka *et al.*, 1998; Brunner & Bernasconi, 2005; Johnston *et al.*, 2005; Canfield *et al.*, 2006; Farquhar *et al.*, 2007) and has been reviewed in detail in several recent studies (Comstock, 2001; Hayes, 2001; Fry, 2003).

The biochemical pathways for Mo uptake, storage, and enzymatic incorporation can be represented by the following simple reaction network:


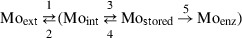
(3)

where the parentheses represent the cell wall, Mo_ext_ is the external (media) molybdate pool, Mo_int_ is the internal molybdate pool, Mo_stored_ is the pool of Mo bound to storage proteins (e.g., MoO_4_^2−^ bound to Mop proteins in freshwater and coastal cyanobacteria; Thiel *et al.*, 2002; Glass *et al.*, 2010), and Mo_enz_ is the pool of Mo bound to enzymes (nitrate reductase or dinitrogenase in this case). Each numbered arrow represents the flow of Mo from one pool to another, and fractionation of isotopes between the two pools can occur along each of the pathways (with a fractionation factor, *α*). In this model, pathway 1 represents transport of Mo into the cell, pathway 2 represents the loss of Mo from the cell, pathway 3 represents binding of Mo to storage proteins, pathway 4 represents release of Mo from storage proteins, and pathway 5 represents incorporation of Mo into enzymes. In this model we assume a steady-state, whereby the isotope values of Mo inside the cell are set by the relative proportions of Mo uptake, loss from the cell, storage, and incorporation into enzymes. The Mo isotope values for stored Mo and enzymatically-incorporated Mo can be calculated with a series of mass balance equations, as presented in the Supporting information.

The fractionations associated with each of these pathways have not been directly measured. However, we can make some estimates based on theoretical models of isotope effects produced during coordination changes in Mo species. Tossel (2005) used quantum mechanical calculations to estimate isotope fractionation equilibrium constants for a number of Mo compounds, suggesting fractionation factors of 0.9979–0.9985 between tetrahedrally-bound Mo in MoO_4_^2−^ to octahedrally-bound Mo in Mo(OH)_6_. We used the mean from these calculations to approximate a fractionation factor of *α*_5_ = 0.9982 for the coordination change associated with binding of Mo from tetrahedrally-coordinated molybdate into the nitrate reductase and dinitrogenase enzymes, where it is bound in octahedral coordination (Burgess & Lowe, 1996; Hille, 1996; Moura *et al.*, 2004). Both ModA (the periplasmic Mo-binding protein) and Mop (the Mo-binding storage protein found in some coastal and freshwater cyanobacteria) bind Mo as molybdate, without a change in coordination (Wagner *et al.*, 2000; Thiel *et al.*, 2002; Schüttelkopf *et al.*, 2003; Zahalak *et al.*, 2004; Masters *et al.*, 2005). We therefore initially assumed no fractionation during Mo transport into and out of the cell or during storage and release of molybdate (*α*_1_, *α*_2_, *α*_3_, *α*_4_ = 1). We tested the impact of including fractionations during Mo uptake and storage, as discussed below. Model parameters and assumptions are listed in the Supporting information.

**Fig. 3:**
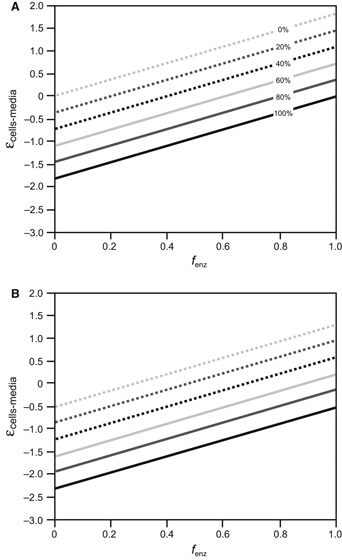
Values calculated with a reaction network model for *ɛ*_cells–media_ versus *f*_enz,_ as defined in the text (see text and Supporting information for derivation of model and model parameters). The contours represent the proportion of total cellular Mo (the quantity analyzed) that was enzyme-bound (assuming total cellular Mo = enzyme-bound Mo + Mo present in storage proteins). (A) Illustrates a model with no fractionation during Mo transport, (B) is a model including a 0.5‰ fractionation during Mo transport (see text and Supporting information).

The results of the model are illustrated in Fig. 3, plotted as *ɛ*_cells–media_ vs. the proportion of the stored Mo pool that is incorporated into enzymes ( *f*_enz_, numerically representing the relative mass flow of Mo along pathway 5 compared to mass flow of Mo along pathway 5 + pathway 4; see Supporting information). The contours on the plots represent the distribution of the measured cellular Mo between enzymes and storage proteins, such that at the 100% contour all of the cellular Mo is in enzymes, and at the 0% contour all of the cellular Mo is in storage proteins. This figure demonstrates how transport and storage, enzymatic incorporation, and the intracellular distribution of Mo could all contribute to biological fractionations. The fractionation factor associated with Mo uptake, *α*_1_, sets the upper limit for 100% incorporation of stored Mo into enzymes (set to 1 in Fig. 3A and at 0.9995 in Fig. 3B). This is because if the stored Mo pool is quantitatively incorporated into enzymes, then no isotopic fractionation is expressed from the enzymatic incorporation step. As smaller proportions of the stored Mo pool are enzymatically incorporated ( *f*_enz_), expression of the fractionation associated with the incorporation step increases, and as a consequence the cumulative fractionation is larger. The fractionation factor associated with storage of Mo, *α*_3_, has a similar effect on the model as *α*_1_, and in the absence of a fractionation during uptake will define the upper bound for *f*_enz_ = 1. If fractionations are assigned for both uptake and storage, the predicted cumulative fractionations are much larger (resulting in cellular δ^98^Mo values down to −2.8‰ for *α*_1_ and *α*_3_ values set to 0.9995; not shown).

This model could explain the difference in fractionations between nitrate-utilizing and N_2_-fixing experiments, as well as the change in fractionations with growth stage during N_2_ fixation. In this scenario, larger fractionations would be generated during N_2_ fixation than during nitrate utilization because a smaller proportion of the stored Mo would be incorporated into enzymes during N_2_ fixation than during nitrate utilization. Likewise, more of the stored Mo pool would be incorporated into enzymes during stationary phase than during exponential growth when fixing N_2_. This result may seem counter-intuitive; however, at steady state *f*_enz_ is independent of the size of the stored Mo pool, and therefore these results could simply indicate an increase in the storage of Mo during N_2_ fixation and during exponential growth. This result is consistent with a higher cellular Mo content in *A. variabilis* during N_2_ fixation, and with high levels of Mo storage during N_2_ fixation estimated for another freshwater heterocystous cyanobacterium, *Nostoc* sp. PCC 7120 (Glass *et al.*, 2010).

This exercise demonstrates how a metabolic model can be utilized to constrain fractionation processes and examine the flow of Mo through bacterial metabolisms. However, significant questions remain about the fractionations associated with Mo transport and storage in this and other organisms. For example, *Azotobacter vinelandii* utilizes a ModA periplasmic Mo-binding protein that is less similar to the Mo-binding proteins of freshwater and marine cyanobacteria (Zahalak *et al.*, 2004; Fig. 1). This organism also has a rare Mo storage system (MoSto), which stores Mo as Mo-oxide aggregates (Pienkos & Brill, 1981; Fenske *et al.*, 2005; Schemberg *et al.*, 2007, 2008), rather than as molybdate in the Mop system (Wagner *et al.*, 2000; Schüttelkopf *et al.*, 2003; Masters *et al.*, 2005). Changes in fractionations associated with uptake and storage by these different systems could account for the differences in fractionations between *A. variabilis* and *Azotobacter vinelandii* (Table 1). Additional measurements of fractionations produced during Mo uptake in other organisms (e.g., marine cyanobacteria) and under variable environmental conditions (e.g., at lowered Mo concentrations) will test this model and inform future models, as will a more detailed understanding of the biochemistry of Mo uptake and storage in cyanobacteria.

## Geobiological Significance

**Fig. 4:**
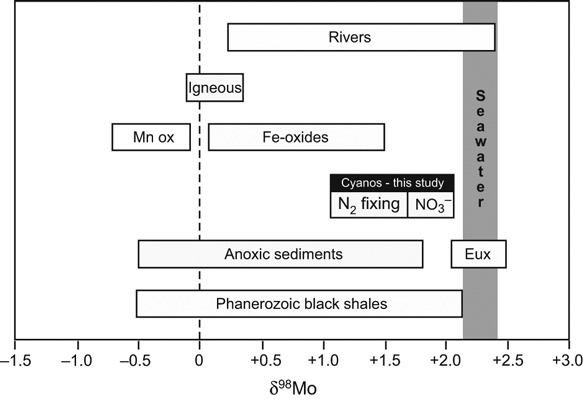
The δ^98^Mo values measured in some natural samples (from Barling *et al.*, 2001; Siebert *et al.*, 2003; Arnold *et al.*, 2004; Barling & Anbar, 2004; Nägler *et al.*, 2005; Poulson *et al.*, 2006; Siebert *et al.*, 2006; Archer & Vance, 2008; Nakagawa *et al.*, 2008; Wasylenki *et al.*, 2008; Goldberg *et al.*, 2009; Gordon *et al.*, 2009; this study). Values from this study include cellular Mo measured in cyanobacteria utilizing nitrate (NO_3_^−^) and fixing N_2_ (N fix). Anoxic and Eux (euxinic) values are from modern sediments deposited under anoxic conditions, either with sulfide confined to porewaters (anoxic) or with free sulfide in the water column (euxinic). Experimental results (for Mo sorbed to Mn- and Fe-oxides and for cellular Mo from this study) were normalized to modern seawater values to reflect the range in δ^98^Mo that might be expected in natural systems.

We have demonstrated that cyanobacterial assimilation of Mo can produce large fractionations in δ^98^Mo (*ɛ*_cells–media_ as large as −1‰), particularly during growth when nitrogen is the only limiting nutrient (such as could occur during bloom events in natural systems). These fractionations are comparable to those produced by other sedimentary processes, and could produce δ^98^Mo values that overlap with those of Mo in sedimentary organic matter deposited in anoxic settings (Fig. 4). Marine N_2_-fixing cyanobacteria utilize proteins homologous to the freshwater organism tested here for Mo uptake (Fig. 1) and N_2_ fixation (Dominic *et al.*, 2000), though Mo storage proteins such as Mop have not been found in marine cyanobacteria (see review in Glass *et al.*, 2010). Unless the primary fractionation is associated with storage and release of Mo from the Mop protein (which is unlikely to be the case because it binds Mo as molybdate, see Discussion above), these organisms should be able to produce fractionations of a similar magnitude. If this is the case, then N_2_-fixing cyanobacteria could provide an important source of isotopically-light Mo bound to organic matter in sedimentary environments, particularly in anoxic (non-sulfidic) settings. The spatial distribution of marine N_2_ fixation is tightly coupled to anoxic regions of N loss via nitrification and anaerobic oxidation of ammonia (anammox) (Deutsch *et al.*, 2007). Nitrogen fixation in the modern oceans appears to be enhanced in surface waters above oxygen minimum zones, such as above the eastern tropical north Pacific and in the Arabian Sea (Brandes *et al.*, 1998; Deutsch *et al.*, 2007). The smaller fractionations associated with nitrate uptake and very slow growth (i.e., stationary phase) during N_2_ fixation might then be more common in open-ocean environments, where organisms are frequently faced with starvation conditions and enter intermittent periods of no growth or very slow growth.

Molybdenum associated with N_2_-fixing cyanobacteria could have provided a source of ^98^Mo-depleted Mo in ancient sedimentary organic matter as well. Cyanobacteria likely developed a biochemical mechanism for Mo utilization and N_2_ fixation similar to that of modern organisms very early in Earth history (Glass *et al.*, 2009). Dinitrogenase is found in diverse micro-organisms distributed across both prokaryotic domains (Young, 1992; Zehr *et al.*, 2000; Zehr & Turner, 2001), and shows a high degree of conservation of structure, function, and amino acid sequence (Dean & Jacobson, 1992), indicating an ancient origin (Raymond *et al.*, 2004). Some researchers have suggested that the alternative dinitrogenases preceded the Mo-containing enzyme in ancient Mo-depleted oceans (Anbar & Knoll, 2002; Raymond *et al.*, 2004; Glass *et al.*, 2009). Experimental investigations of Mo requirements during N_2_ fixation indicate that nitrogen fixation rates in organisms utilizing the Fe-Mo dinitrogenase are only hindered at Mo concentrations lower than about 5% of modern marine concentrations (Zerkle *et al.*, 2006; Glass *et al.*, 2010). Recent studies of Mo in black shales indicates that the marine Mo reservoir could have been as large as 10–20% of that of the modern ocean, making Mo-dependent nitrogen fixation a feasible process as early as 2.2 Ga (Scott *et al.*, 2008). Alternatively, the Mo dinitrogenase could have evolved in association with enhanced delivery of Mo to the oceans during transient oxygenation events as early as ∼2.5 Ga (e.g., Anbar *et al.*, 2007).

Certainly the biochemistry of N_2_ fixation and Mo utilization would have been well-established by ∼551 million years ago, when atmospheric O_2_ was near modern levels and Mo concentrations in the oceans were likely similar to that of today (Scott *et al.*, 2008). Studies of carbon and nitrogen isotopes of organic matter and biomarkers in numerous black shales deposited during expanded periods of ocean anoxia during the Phanerozoic (termed oceanic anoxic events, OAEs) indicate that N_2_-fixing cyanobacteria were the primary contributors of organic matter in these sediments (Chicarelli *et al.*, 1993; Ohkouchi *et al.*, 1997, 2006; Sachs & Repeta, 1999; Kuypers *et al.*, 2004; Junium & Arthur, 2007; Karakitsios *et al.*, 2007; Kashiyama *et al.*, 2008; Meyers *et al.*, 2009). N_2_ fixation was presumably enhanced during Phanerozoic OAEs because of the near complete removal of fixed N by denitrification and anammox along with increased phosphate availability from preferential release of P from anoxic sediments (Sachs & Repeta, 1999; Kuypers *et al.*, 2004; Junium & Arthur, 2007). Some, but not all, of these black shales exhibit evidence of euxinic water column conditions during deposition (Sinninghe Damste & Koester, 1998; Joachimski *et al.*, 2001; Pancost *et al.*, 2004). Other Phanerozoic shales show evidence for deposition under suboxic, or under intermittently euxinic to suboxic conditions (e.g., Gordon *et al.*, 2009). The fractionations in δ^98^Mo we measure during Mo uptake in N_2_-fixing cyanobacteria overlap with fractionations observed in modern anoxic (non-sulfidic) systems and in anoxic Phanerozoic sediments (Fig. 4). This correlation suggests that cyanobacteria could be an important contributor to this Mo isotope signal, particularly when separate lines of evidence point to a significant organic matter contribution from cyanobacteria fixing N_2_.

## SUPPORTING INFORMATION

**Data S1.**Reaction network model.

**Figure S1.** Alignment length comparisons ofblast hits to*Anabaena variabilis* ModBC. Species name and locus tag foreach homolog are specified. The only proteins that align along thefull length of ModBC from *A. variabilis* are other cyanobacterial protein sequences; homologous open reading frames from other species align with either the ModB or ModC fraction.

**Table S1.**ModA sequences included inalignment. Accession number is for the ModA ortholog in eachspecies. % I/S: % identity to ModA from *A. variabilis*/%similarity to ModA from *A. variabilis*. References aregiven for those ModA proteins that have been genetically,biochemically or structurally characterized.
