# Supplementary material for: Molybdenum isotope fractionation by cyanobacterial assimilation during nitrate utilization and N2fixation
Source: Geobiology. 2011 Jan;9(1):94–106. doi: 10.1111/j.1472-4669.2010.00262.x (PMC3627308; doi:10.1111/j.1472-4669.2010.00262.x)
Supplement: Supplementary file 1 [file gbi0009-0094-SD1.docx]

**SUPPLEMENTARY MATERIAL**

**REACTION NETWORK MODEL**

We constructed a simple mass balance model for Mo flow through the cell following the metabolic network approach reviewed in Hayes (2001), Comstock (2001), and Fry (2003). The biochemical pathways for Mo uptake and incorporation into enzymes can be represented by the simplified network model, as in the text:

(A1)

where the parentheses represent the cell wall, Mo_ext_ is the external Mo pool, Mo_int_ is the internal molybdate pool, Mo_stored_ is the pool of Mo bound to storage proteins, and Mo_enz_ is the pool of Mo bound to enzymes (nitrogenase or nitrate reductase). Each numbered arrow represents the flow of Mo from one pool to another, such that 1 = uptake of Mo into the cell, 2 = flow of Mo out of the cell, 3 = binding of Mo by a storage protein, 4 = unbinding of Mo, and 5 = incorporation of Mo into enzymes. Reversibility of molybdate uptake into the cell (flow 2) has not been examined; however, the sulfate anion has a similar size and polarity to molybdate and a similar ABC transport system, and sulfate transport has been shown to be reversible (Cypionka, 1989). Assuming steady state for any Mo pool, the summation of all mass flows (φ_i_) of Mo into any given pool of Mo is equal to the mass flow out. Therefore we can write mass balance on the stored Mo pool:

$\varphi_{3}=\varphi_{4}+\varphi_{5}$ (A2)

We can also define *f*_5_  as the relative mass flow of Mo along pathway 5 compared to mass flow of Mo along pathway 5 + pathway 4, which represents the proportion of the stored Mo pool that is incorporated into enzymes,

$f_{5}=\frac{\varphi_{5}}{\varphi_{4}+\varphi_{5}}$. (A3)

This quantity is referred to in the text and Figure 3 as *f*_enz_. The isotopic ratio of Mo entering each pool along pathway *i* (as R_i_ = ^98/95^Mo_i_/^98/95^Mo_std_) can be calculated as:

$R_{i}=R_{j}\cdot\alpha_{i}$ (A4)

where R_j_ is the ^98/95^Mo of the starting pool (Mo_int_ or Mo_stored_), and *α*_i_ is the fractionation imparted along each pathway (i = 1, 2, 3, 4 and 5). We can substitute Equations A3 and A4 into Equation A2:

$R_{Mo,\mathrm{int}}\cdot\alpha_{3}=f_{5}\cdot R_{Mo,stored}\cdot\alpha_{5}+(1-f_{5})\cdot R_{Mo,stored}\cdot\alpha_{4}$. (A5)

Rearranging terms,

$\frac{R_{Mo,\mathrm{int}}}{R_{Mo,stored}}=\frac{f_{5}\cdot\alpha_{5}+(1-f_{5})\cdot\alpha_{4}}{\alpha_{3}}$. (A6)

Similarly, by mass balance

$R_{Mo,stored}=\frac{R_{Mo,enz}}{\alpha_{5}}$ (A7)

and

$\frac{R_{Mo,ext}}{R_{Mo,\mathrm{int}}}=\frac{f_{3}\cdot\alpha_{3}+(1-f_{3})\cdot\alpha_{2}-(1-f_{1})\cdot\frac{R_{Mo,stored}}{R_{Mo,\mathrm{int}}}\cdot\alpha_{4}}{f_{1}\cdot\alpha_{1}}$. (A8)

To solve for the fractionation between the media and enzyme-bound Mo, we substitute Equation A6 into Equation A8 to calculate R_Mo,ext_/R_Mo,int_, and plug equation A7 into A6 to solve for R_Mo,int_/R_Mo,enz_ (assuming an external δ^98^Mo of 0‰). We then multiply Equation A8 (R_Mo,ext_/R_Mo,int_ ) by Equation A6 (R_Mo,int_/R_Mo,stored_) to calculate the fractionation between the media and stored Mo.

In the calculations presented in Figure 3 we assumed a fractionation factor of *α*_5_ = 0.9982 for a coordination change from tetrahedral to octahedral coordination during enzymatic incorporation (Tossell, 2005). We assumed fractionation factors of 1 for *α*_2_ and *α*_4_ (flow of Mo out of the cell and release of Mo from storage), and examined sensitivity of the model to fractionation factors for *α*_1_ and *α*_3_ (Mo uptake and Mo storage), as discussed in the text (*α*_1_ = 1 for Figure 3a and *α*_1_ = 0.9995 for Figure 3b). We set *f*_1_ to 1, assuming that all of the free internal Mo pool is from uptake (rather than release from storage). Changing the value for *f*_3_ did not significantly affect the model. We then varied *f*_5_ (the proportion of stored Mo that is incorporated into enzymes) to produce the plots in Figure 3.

Supplementary Table 1. ModA sequences included in alignment. Accession number is for the ModA ortholog in each species. %I/S: % identity to ModA from *A. variabilis*/% similarity to ModA from *A. variabilis*. References are given for those ModA proteins that have been genetically, biochemically or structurally characterized.

| **Species** | **Group** | **NCBI accession number** | **%I/S** | **Reference** |
| --- | --- | --- | --- | --- |
| Candidatus “Koribacter versatilis” | Acidobacteria | YP_590749.1 | 31/49 |  |
| *Arthrobacter nicotinivorans* | Actinobacteria | CAA71776.1 | 34/47 | Menendez et al., 1997 |
| *Kineococcus radiotolerans* SRS30216 | Actinobacteria | YP_001363904.1 | 30/46 |  |
| *Mycobacterium tuberculosis* KZN 1435 | Actinobacteria | YP_003032100.1 | 28/47 |  |
| *Streptomyces coelicolor* A3(2) | Actinobacteria | NP_627896.1 | 30/47 |  |
| *Bradyrhizobium japonicum* | Alphaproteobacteria | AF446208_2 | 38/53 | Delgado et al., 2006 |
| *Caulobacter crescentus* NA1000 | Alphaproteobacteria | YP_002515706.1 | 30/50 |  |
| *Rhodobacter capsulatus* | Alphaproteobacteria | Q08383.1 | 27/46 | Wang et al., 1993 |
| *Rhodopseudomonas palustris* BisB5 | Alphaproteobacteria | YP_568102.1 | 31/50 |  |
| *Rhodospirillum rubrum* ATCC 11170 | Alphaproteobacteria | YP_425795.1 | 37/55 |  |
| *Aquifex aeolicus* VF5 | Aquificae | NP_214105.1 | 32/49 |  |
| *Hydrogenobacter thermophilus* TK-6 | Aquificae | YP_003432974.1 | 34/52 |  |
| *Persephonella marina* EX-H1 | Aquificae | YP_002731679.1 | 28/48 |  |
| *Cupriavidus taiwanensis* str. LMG19424 | Betaproteobacteria | YP_002004684.1 | 37/56 |  |
| *Polaromonas naphthalenivorans* CJ2 | Betaproteobacteria | YP_982267.1 | 26/48 |  |
| *Thauera* sp. MZ1T | Betaproteobacteria | YP_002354205.1 | 29/46 |  |
| *Thiomonas intermedia* K12 | Betaproteobacteria | YP_003641950.1 | 28/46 |  |
| *Chlorobium ferrooxidans DSM 13031* | Chlorobi | EAT59909.1 | 36/52 |  |
| *Chlorobium tepidum* TLS | Chlorobi | NP_662428.1 | 30/51 |  |
| “Nostoc azollae” 0708 | Cyanobacteria | YP_003720061.1 | 71/83 |  |
| *Acaryochloris marina* MBIC11017 | Cyanobacteria | YP_001515131.1 | 44/68 |  |
| *Anabaena variabilis* | Cyanobacteria | YP_322936.1 | --/-- | Zahalak et al. , 2004 |
| Cyanobacterium UCYN-A | Cyanobacteria | YP_003421195.1 | 45/65 |  |
| *Cyanothece* sp. ATCC 51142 | Cyanobacteria | YP_001802264.1 | 53/70 |  |
| *Cyanothece* sp. PCC 7424 | Cyanobacteria | YP_002380213.1 | 53/69 |  |
| *Microcystis aeruginosa* NIES-843 | Cyanobacteria | YP_001660559.1 | 56/74 |  |
| *Nostoc punctiforme* sp. PCC 73012, chromosomal copy | Cyanobacteria | YP_001865940.1 | 71/86 |  |
| *Nostoc punctiforme* sp. PCC 73012, plasmid copy | Cyanobacteria | YP_001870044.1 | 31/53 |  |
| *Nostoc* sp. PCC 7120 | Cyanobacteria | NP_489218.1 | 94/97 |  |
| *Synechococcus* sp. JA-2-3B’a(2-13) | Cyanobacteria | YP_476658.1 | 41/64 |  |
| *Synechocystis* sp. PCC 6803 | Cyanobacteria | NP_442706.1 | 48/67 |  |
| *Trichodesmium erythraeum* IMS101 | Cyanobacteria | YP_723628.1 | 47/71 |  |
| *Arcobacter nitrofigilis* DSM 7299 | Epsilonproteobacteria | YP_003654220.1 | 24/47 |  |
| *Campylobacter jejuni* subsp. *jejuni* NCTC 11168 | Epsilonproteobacteria | YP_002343741.1 | 27/47 | Smart et al., 2009 |
| *Helicobacter pylori* P12 | Epsilonproteobacteria | YP_002301117.1 | 31/51 |  |
| *Bacillus cereus* AH187 | Firmicutes | YP_002336315.1 | 49/68 |  |
| *Bacillus subtilis* subsp. *subtilis* | Firmicutes | NP_391219.2 | 47/64 |  |
| *Clostridium botulinum* B1 str. Okra | Firmicutes | YP_001781120.1 | 43/64 |  |
| *Geobacillus kaustophilus* HTA426 | Firmicutes | YP_148557.1 | 50/65 |  |
| *Geobacillus* sp. Y412MC10 | Firmicutes | YP_003243513.1 | 46/62 |  |
| *Staphylococcus aureus* subsp. *aureus* ST398 | Firmicutes | SAPIG2330 | 40/60 |  |
| *Staphylococcus carnosus* subsp. *carnosus* TM300 | Firmicutes | YP_002634858.1 | 41/59 |  |
| *Azotobacter vinelandii* | Gammaproteobacteria | CAA48819.1 | 28/45 | Luque et al., 1993 |
| *Escherichia coli* (sulfate-binding protein) | Gammaproteobacteria | CAA26357.1 | 25/41 | Hellinga and Evans, 1985 |
| *Escherichia coli* K-12 substr MG1655 | Gammaproteobacteria | NP_415284.1 | 37/54 | Miyake et al., 1995 |
| *Haemophilus influenzae* 10810 | Gammaproteobacteria | CBW30055.1 | 37/57 |  |
| *Pantoea ananatis* LMG 20103 | Gammaproteobacteria | YP_003519502.1 | 39/55 |  |
| *Pseudomonas aeruginosa* PA7 | Gammaproteobacteria | YP_001348787.1 | 28/47 |  |
| *Shewanella baltica* OS195 | Gammaproteobacteria | YP_001553268.1 | 41/58 |  |
| *Shewanella oneidensis* MR-1 | Gammaproteobacteria | NP_719395.1 | 40/57 |  |
| *Xanthomonas axonopodis* pv. citri str. 306 | Gammaproteobacteria | NP_643665.1 | 35/52 | Santacruz et al., 2008 |
| *Blastopirellula marina* DSM 3645 | Planctomycetes | ZP_01092119.1 | 35/55 |  |
| *Pirellula staleyi* DSM 6068 | Planctomycetes | YP_003373019.1 | 39/61 |  |

**FIGURE CAPTIONS**

Supplementary Figure 1. Alignment length comparisons of BLAST hits to *Anabaena variabilis* ModBC. Species name and locus tag for each homolog are specified. The only proteins that align along the full length of ModBC from *A. variabilis* are other cyanobacterial protein sequences; homologous open reading frames from other species align with either the ModB or ModC fraction.

**REFERENCES**

Comstock JP (2001) Steady-state isotopic fractionation in branched pathways using plant uptake of NO_3_^-^ as an example. *Planta,* **214**, 220-234.

Cypionka H (1989) Characterization of sulfate transport in *Desulfovibrio desulfuricans*. *Archives of Microbiology,* **152**, 237-243.

Delgado MJ, Tresierra-Ayala A, Talbi C, Bedmar EJ (2006) Functional characterization of the *Bradyrhizobium japonicum modA* and *modB* genes involved in molybdenum transport. *Microbiology,* **152**, 199-207.

Fry B (2003) Steady state models of stable isotope distributions. *Isotopes in Environmental Health Studies,* **39**, 219-232.

Hayes JM (2001) Fractionation of carbon and hydrogen isotopes in biosynthetic processes. In: *Stable Isotope Geochemistry* (eds Valley Jw, Cole Dr). Mineralogical Society of American, Blacksburg, VA*,* pp. 225-277.

Hellinga HW, Evans PR (1985) Nucleotide sequence and high-level expression of the major *Escherichia coli* phosphofructokinase. *European Journal of Biochemistry,* **149**, 363-373.

Luque F, Mitchell LA, Chapman M, Christine R, Pau RN (1993) Characterization of genes involved in molybdenum transport in *Azotobacter vinelandii*. *Molecular Microbiology,* **7**, 447-459.

Menendez C, Otto A, Igloi G, Nick P, Brandsch R, Schubach B, Bottcher B, Brandsch R (1997) Molybdate-uptake genes and molybdopterin-biosynthesis genes on a bacterial plasmid -- characterization of MoeA as a filament-forming protein with adenosinetriphosphatase activity. *European Journal of Biochemistry,* **250**, 524-523.

Miyake H, Yabu H, Satoh H, Yamamoto I (1995) Characterization and transcriptional regulation of the modABCD genes for molybdenum transport in *Escherichia coli*. *Nucleic Acids Symposium Series,* **1995**, 91-92.

Santacruz CP, Balan A, Ferreira LC, Barbosa JA (2006) Crystallization, data collection and phasing of the molybdate-binding protein of the phytopathogen *Xanthomonas axonopodis* pv. citri. *Acta Crystallographica Section F: Structural Biology and Crystallization Communications,* **62**, 289-291.

Smart JP, Cliff MJ, Kelly DJ (2009) A role for tungsten in the biology of Campylobacter jejuni: tungstate stimulates formate dehydrogenase activity and is transported via an ultra-high affinity ABC system distinct from the molybdate transporter. *Molecular Microbiology,* **74**, 742-757.

Tossel JA (2005) Calculating the partitioning of the isotopes of Mo between oxic and sulfidic species in aqueous solutions. *Geochimica et Cosmochimica Acta,* **69**, 2981-2993.

Wang G, Angermuller S, Klipp W (1993) Characterization of Rhodobacter capsulatus genes encoding a molybdenum transport system and putative molybdenum-pterin-binding proteins. *Journal of Bacteriology,* **175**, 3031-3042.

Zahalak M, Pratte B, Werth KJ, Thiel T (2004) Molybdate transport and its effect on nitrogen utilization in the cyanobacterium *Anabaena variabilis* ATCC 29413. *Molecular Microbiology,* **51**, 539-549.
