# Supplementary figures and images for: Molybdenum isotope fractionation by cyanobacterial assimilation during nitrate utilization and N2fixation
Source: Geobiology. 2011 Jan;9(1):94–106. doi: 10.1111/j.1472-4669.2010.00262.x (PMC3627308; doi:10.1111/j.1472-4669.2010.00262.x)

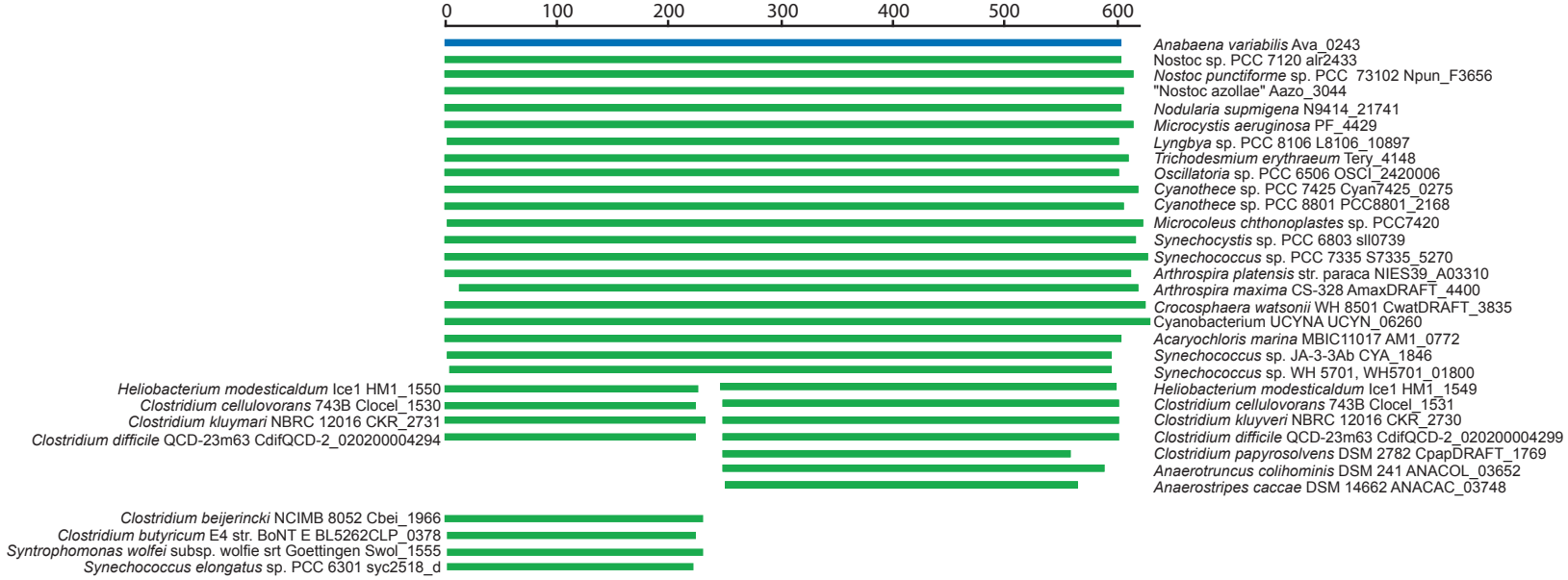

Supplement: Supplementary file 2 [file gbi0009-0094-SD2.pdf]
